# Redetermination of 5-iodo­uracil

**DOI:** 10.1107/S1600536807068043

**Published:** 2008-01-04

**Authors:** Gustavo Portalone

**Affiliations:** a Chemistry Department, ’Sapienza’ University of Rome, P. le A. Moro, 5, I-00185 Rome, Italy

## Abstract

The title compound (systematic name: 2,4-dihydr­oxy-5-iodo­pyrimidine), C_4_H_3_IN_2_O_2_, which was first reported by Sternglanz, Freeman & Bugg [*Acta Cryst.* (1975 ▶), B**31**, 1393–1395], has been redetermined, providing a significant increase in the precision of the derived geometric parameters. The asymmetric unit comprises a non-planar mol­ecule in a slightly distorted B_25_ boat conformation. The mol­ecules are associated in the crystal structure to form ribbons stabilized by N—H⋯O hydrogen bonds which involve NH groups and two carbonyl O atoms.

## Related literature

For the previous structure determination, see: Sternglanz *et al.* (1975 ▶). For a general approach to the use of multiple-hydrogen-bonding DNA/RNA nucleobases as potential supra­molecular reagents, see: Portalone *et al.* (1999 ▶); Brunetti *et al.* (2000 ▶, 2002 ▶); Portalone & Colapietro (2007 ▶, and references therein). For computation of ring patterns formed by hydrogen bonds in crystal structures, see: Etter *et al.* (1990 ▶); Bernstein *et al.* (1995 ▶); Motherwell *et al.* (1999 ▶). the B_25_ boat confromation is defined by Cremer & Pople (1975 ▶).

For related literature, see: Portalone *et al.* (2002 ▶).
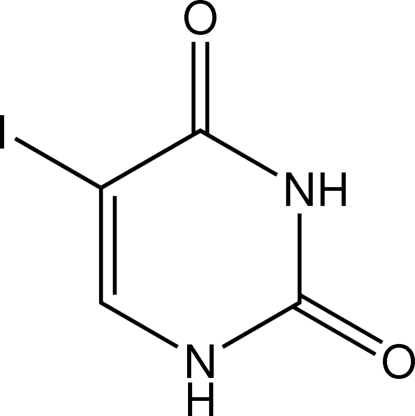


## Experimental

### 

#### Crystal data

C_4_H_3_IN_2_O_2_
*M* *_r_* = 237.98
Monoclinic, 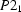

*a* = 4.89650 (18) Å
*b* = 4.45921 (13) Å
*c* = 14.2167 (2) Å
β = 92.341 (2)°
*V* = 310.16 (1) Å^3^
*Z* = 2
Mo *K*α radiation
μ = 5.08 mm^−1^
*T* = 298 (2) K
0.40 × 0.20 × 0.10 mm

#### Data collection

Oxford Diffraction Xcalibur S CCD diffractometer
Absorption correction: multi-scan (*SCALE3 ABSPACK*; Oxford Diffraction, 2006 ▶).*T*_min_ = 0.252, *T*_max_ = 0.602
48636 measured reflections
2127 independent reflections
1803 reflections with *I* > 2σ(*I*)
*R*_int_ = 0.049

#### Refinement

*R*[*F* ^2^ > 2σ(*F* ^2^)] = 0.025
*wR*(*F* ^2^) = 0.071
*S* = 1.04
2127 reflections
86 parameters
1 restraint
H-atom parameters constrained
Δρ_max_ = 0.38 e Å^−3^
Δρ_min_ = −0.03 e Å^−3^
Absolute structure: Flack (1983 ▶), 934 Friedel pairs
Flack parameter: −0.01 (2)

### 

Data collection: *CrysAlis CCD* (Oxford Diffraction, 2006 ▶); cell refinement: *CrysAlis RED* (Oxford Diffraction, 2006 ▶); data reduction: *CrysAlis RED*; program(s) used to solve structure: *SIR97* (Altomare *et al.*, 1999 ▶); program(s) used to refine structure: *SHELXL97* (Sheldrick, 1997 ▶); molecular graphics: *WinGX* (Farrugia, 1999 ▶); software used to prepare material for publication: *WinGX*.

## Supplementary Material

Crystal structure: contains datablocks global, I. DOI: 10.1107/S1600536807068043/tk2237sup1.cif

Structure factors: contains datablocks I. DOI: 10.1107/S1600536807068043/tk2237Isup2.hkl

Additional supplementary materials:  crystallographic information; 3D view; checkCIF report

## Figures and Tables

**Table 1:** Hydrogen-bond geometry (Å, °)

*D*—H⋯*A*	*D*—H	H⋯*A*	*D*⋯*A*	*D*—H⋯*A*
N1—H1⋯O2^i^	0.88	2.22	2.897 (3)	133
N3—H3⋯O1^ii^	0.86	1.92	2.767 (3)	170

Symmetry codes: (i) 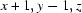; (ii) 
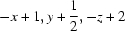
.

**Fig. 1.**
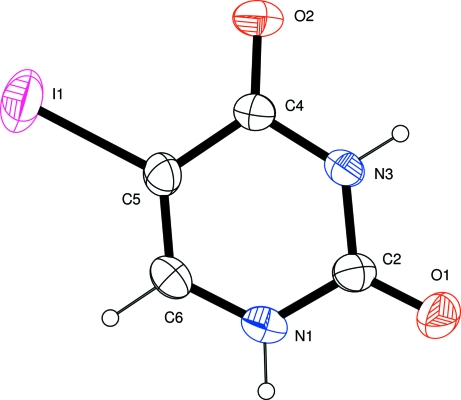
The molecular structure of (I), showing the atom-labelling scheme. Displacements ellipsoids are at the 50% probability level.

**Fig. 2.**
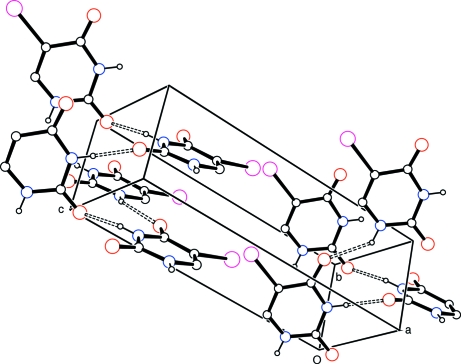
Crystal packing diagram for (I) viewed approximately down c. All atoms are shown as small spheres of arbitrary radii. For the sake of clarity, H atoms not involved in hydrogen bonding have been omitted. Hydrogen bonding is indicated by dashed lines.

## supplementary crystallographic information

### Comment

5-iodouracil, 5IUrac, was determined some 30 years ago (Sternglanz *et al.*, 1975). In this study, 591 unique reflections were collected at ambient temperature on an automatic diffractometer, and the heavy-atom method was employed to solve the crystal structure. Only non-H atoms were localized and refined. The final refinement, carried out on a fairly small data set, led to *R* = 0.044, a data-to-parameter ratio of 7.2, S = 2.52 and standard deviations of 0.018Å in C—C bond lengths and 0.9° in bond angles, As a part of a more general study of multiple-hydrogen-bonding DNA/RNA nucleobases as potential supramolecular reagents (Brunetti *et al.*, 2000, 2002; Portalone *et al.*, 1999; Portalone *et al.*, 2002; Portalone & Colapietro, 2007), this paper reports a redetermination of the crystal structure of the title compound, (I), with greater precision and accuracy. The asymmetric unit of (I) comprises a non-planar independent molecule (Fig. 1) in a slightly distorted B_25_ boat conformation (Cremer & Pople, 1975). Analysis of the crystal packing of (I), (Fig. 2), shows that the structure is stabilized by two intermolecular N—H···O interactions of descriptor C^1^_1_(3) (Etter *et al.*, 1990; Bernstein *et al.*, 1995; Motherwell *et al.*, 1999) (Table 1) between NH moieties and two carbonyl O atoms (O2^i^ and O1^ii^) [symmetry code: (i) *x* + 1, *y* - 1, *z*; (ii) -*x* + 1, *y* + 1/2, -*z* + 2] which link the molecules into ribbons.

### Experimental

The title compound (0.1 mmol, Sigma Aldrich at 98% purity) was dissolved in water (6 ml) and heated under reflux for 1 h. After cooling the solution to ambient temperature, crystals suitable for single-crystal X-ray diffraction were grown by slow evaporation of the solvent.

### Refinement

The H atoms were included in the riding model approximation with C—H = 0.96 Å and N—H = 0.86–0.88 Å, and with refined isotropic displacement parameters.

### Crystal data

**Table:**

C_4_H_3_IN_2_O_2_	*F*_000_ = 220
*M**_r_* = 237.98	*D*_x_ = 2.548 Mg m^−^^3^
Monoclinic, *P*2_1_	Mo *K*α radiation λ = 0.71069 Å
Hall symbol: P 2yb	Cell parameters from 29638 reflections
*a* = 4.89650 (18) Å	θ = 2.9–32.4º
*b* = 4.45921 (13) Å	µ = 5.08 mm^−^^1^
*c* = 14.2167 (2) Å	*T* = 298 (2) K
β = 92.341 (2)º	Tablets, colourless
*V* = 310.157 (15) Å^3^	0.40 × 0.20 × 0.10 mm
*Z* = 2	

### Data collection

**Table:**

Oxford Diffraction Xcalibur S CCD diffractometer	2127 independent reflections
Radiation source: Enhance (Mo) X-ray source	1803 reflections with *I* > 2σ(*I*)
Monochromator: graphite	*R*_int_ = 0.049
Detector resolution: 16.0696 pixels mm^-1^	θ_max_ = 32.4º
*T* = 298(2) K	θ_min_ = 2.9º
ω and φ scans	*h* = −7→7
Absorption correction: multi-scan(CrysAlis RED; Oxford Diffraction, 2006), Empirical absorption correction using spherical harmonics, implemented in SCALE3 ABSPACK scaling algorithm	*k* = −6→6
*T*_min_ = 0.252, *T*_max_ = 0.602	*l* = −21→21
48636 measured reflections	

### Refinement

**Table:**

Refinement on *F*^2^	Hydrogen site location: inferred from neighbouring sites
Least-squares matrix: full	H-atom parameters constrained
*R*[*F*^2^ > 2σ(*F*^2^)] = 0.025	*w* = 1/[σ^2^(*F*_o_^2^) + (0.0471*P*)^2^ + 0.0442*P*] where *P* = (*F*_o_^2^ + 2*F*_c_^2^)/3
*wR*(*F*^2^) = 0.071	(Δ/σ)_max_ = 0.002
*S* = 1.04	Δρ_max_ = 0.38 e Å^−^^3^
2127 reflections	Δρ_min_ = −0.03 e Å^−^^3^
86 parameters	Extinction correction: none
1 restraint	Absolute structure: Flack (1983), 934 Friedel pairs
Primary atom site location: structure-invariant direct methods	Flack parameter: −0.01 (2)
Secondary atom site location: difference Fourier map	

### Special details

**Table:**

Geometry. All e.s.d.'s (except the e.s.d. in the dihedral angle between two l.s. planes) are estimated using the full covariance matrix. The cell e.s.d.'s are taken into account individually in the estimation of e.s.d.'s in distances, angles and torsion angles; correlations between e.s.d.'s in cell parameters are only used when they are defined by crystal symmetry. An approximate (isotropic) treatment of cell e.s.d.'s is used for estimating e.s.d.'s involving l.s. planes.
Refinement. Refinement of *F*^2^ against ALL reflections. The weighted *R*-factor *wR* and goodness of fit *S* are based on *F*^2^, conventional *R*-factors *R* are based on *F*, with *F* set to zero for negative *F*^2^. The threshold expression of *F*^2^ > σ(*F*^2^) is used only for calculating *R*-factors(gt) *etc*. and is not relevant to the choice of reflections for refinement. *R*-factors based on *F*^2^ are statistically about twice as large as those based on *F*, and *R*- factors based on ALL data will be even larger.

### Fractional atomic coordinates and isotropic or equivalent isotropic displacement parameters (Å^2^)

**Table:**

	*x*	*y*	*z*	*U*_iso_*/*U*_eq_	
I1	0.75060 (5)	0.9092	0.595431 (10)	0.05858 (9)	
O1	0.7917 (3)	0.3946 (8)	0.99376 (14)	0.0427 (4)	
O2	0.4156 (4)	1.0571 (5)	0.78388 (17)	0.0399 (4)	
N1	0.9717 (4)	0.4223 (8)	0.84883 (14)	0.0309 (3)	
H1	1.097	0.289	0.8642	0.037 (9)*	
C2	0.7939 (5)	0.5077 (6)	0.91511 (19)	0.0295 (4)	
N3	0.6128 (4)	0.7269 (5)	0.88650 (15)	0.0291 (4)	
H3	0.5025	0.7887	0.9279	0.058 (12)*	
C4	0.5871 (4)	0.8604 (5)	0.79868 (16)	0.0279 (5)	
C5	0.7757 (5)	0.7440 (6)	0.73108 (17)	0.0310 (4)	
C6	0.9626 (5)	0.5361 (6)	0.75921 (19)	0.0310 (4)	
H6	1.0922	0.4663	0.7153	0.032 (8)*	

### Atomic displacement parameters (Å^2^)

**Table:**

	*U*^11^	*U*^22^	*U*^33^	*U*^12^	*U*^13^	*U*^23^
I1	0.09427 (18)	0.05428 (13)	0.02713 (9)	0.00258 (15)	0.00173 (8)	0.00149 (12)
O1	0.0349 (8)	0.0491 (11)	0.0446 (9)	0.0025 (12)	0.0084 (6)	0.0184 (14)
O2	0.0367 (10)	0.0376 (10)	0.0451 (11)	0.0138 (8)	−0.0008 (8)	0.0023 (8)
N1	0.0250 (7)	0.0282 (8)	0.0396 (9)	0.0038 (11)	0.0016 (6)	−0.0020 (13)
C2	0.0226 (9)	0.0279 (9)	0.0381 (12)	−0.0016 (7)	0.0017 (8)	0.0041 (8)
N3	0.0260 (9)	0.0294 (9)	0.0322 (10)	0.0052 (8)	0.0058 (7)	−0.0008 (8)
C4	0.0255 (9)	0.0272 (14)	0.0308 (9)	0.0021 (8)	−0.0004 (7)	−0.0011 (8)
C5	0.0370 (11)	0.0305 (12)	0.0254 (10)	0.0009 (9)	0.0026 (8)	−0.0041 (8)
C6	0.0295 (10)	0.0306 (10)	0.0333 (11)	0.0000 (8)	0.0042 (8)	−0.0084 (9)

### Geometric parameters (Å, °)

**Table:**

I1—C5	2.063 (2)	C2—N3	1.370 (3)
O1—C2	1.227 (3)	N3—C4	1.384 (3)
O2—C4	1.226 (3)	N3—H3	0.8600
N1—C2	1.363 (3)	C4—C5	1.456 (3)
N1—C6	1.370 (4)	C5—C6	1.351 (4)
N1—H1	0.8762	C6—H6	0.9600
			
C2—N1—C6	122.8 (3)	O2—C4—N3	119.9 (2)
C2—N1—H1	118.6	O2—C4—C5	126.2 (2)
C6—N1—H1	118.6	N3—C4—C5	113.9 (2)
O1—C2—N1	123.0 (3)	C6—C5—C4	119.3 (2)
O1—C2—N3	122.3 (3)	C6—C5—I1	122.37 (19)
N1—C2—N3	114.7 (2)	C4—C5—I1	118.33 (17)
C2—N3—C4	127.5 (2)	C5—C6—N1	121.7 (2)
C2—N3—H3	116.3	C5—C6—H6	119.2
C4—N3—H3	116.3	N1—C6—H6	119.2
			
C6—N1—C2—O1	175.9 (3)	N3—C4—C5—C6	−3.4 (3)
C6—N1—C2—N3	−2.9 (4)	O2—C4—C5—I1	−2.2 (3)
O1—C2—N3—C4	−176.7 (3)	N3—C4—C5—I1	177.51 (16)
N1—C2—N3—C4	2.1 (4)	C4—C5—C6—N1	2.8 (4)
C2—N3—C4—O2	−179.3 (2)	I1—C5—C6—N1	−178.1 (2)
C2—N3—C4—C5	0.9 (3)	C2—N1—C6—C5	0.5 (4)
O2—C4—C5—C6	176.9 (3)		

### Hydrogen-bond geometry (Å, °)

**Table:**

*D*—H···*A*	*D*—H	H···*A*	*D*···*A*	*D*—H···*A*
N1—H1···O2^i^	0.88	2.22	2.897 (3)	133
N3—H3···O1^ii^	0.86	1.92	2.767 (3)	170

Symmetry codes: (i) *x*+1, *y*−1, *z*; (ii) −*x*+1, *y*+1/2, −*z*+2.
